# Association of obstructive sleep apnea and diurnal variation of cystatin C

**DOI:** 10.1186/s12882-024-03472-7

**Published:** 2024-01-29

**Authors:** Seolhyun Lee, Sungryong Noh, Woo Hyun Lee

**Affiliations:** 1 Department of Medicine, Stanford University, Palo Alto, CA USA; 2 Division of Pulmonary & Critical Care, Sleep Medicine, McCovern Medical School, University of Texas at Houston, Houston, TX USA; 3 Department of Otolaryngology, Kangwon National University, Chuncheon, Korea; 4 Departments of Otolaryngology, Kangwon National University Hospital, 156, Baengnyeong-Ro, Chuncheon-Si, Gangwon-Do 24289 Republic of Korea

**Keywords:** Obstructive sleep apnea, Cystatin C, Diurnal variation, Circadian rhythm, Nocturnal hypertension, Chronic kidney disease

## Abstract

**Purpose:**

Obstructive sleep apnea is a known risk factor for the progression of chronic kidney disease. To find early signs of the progression in subjects with obstructive sleep apnea., we assessed the diurnal variation of kidney biomarkers.

**Methods:**

A prospective observational study was conducted at Kangwon National University Hospital, Chuncheon, South Korea. All participants underwent in-laboratory polysomnography and phlebotomy in the evening before the polysomnography and in the morning after the polysomnography. Kidney biomarkers, including serum creatinine, blood urea nitrogen, and serum cystatin C, were measured. Delta kidney biomarkers were calculated by subtracting the evening level of the biomarkers from the morning level.

**Results:**

Twenty-six of 50 participants had severe obstructive sleep apnea. Delta cystatin C was significantly correlated with apnea–hypopnea index, oxygen desaturation index, and total arousal index with coefficients of -0.314, -0.323, and -0.289, respectively. In participants without severe obstructive sleep apnea, the morning cystatin C level (0.84 ± 0.11 mg/L) was significantly higher than the evening cystatin C level (0.81 ± 0.11 mg/L) (*P* = 0.005). With severe obstructive sleep apnea, the cystatin C levels were not different between the morning (0.85 ± 0.11 mg/L) and the evening (0.85 ± 0.10 mg/L).

**Conclusions:**

Cystatin C level was increased in the morning in participants without severe obstructive sleep apnea, but not in participants with severe obstructive sleep apnea.

**Supplementary Information:**

The online version contains supplementary material available at 10.1186/s12882-024-03472-7.

## Introduction

Obstructive sleep apnea (OSA) is characterized by recurrent upper airway collapse associated with intermittent hypoxia during sleep. OSA is a prevalent disease and estimated to affect 20% of the adult population. It is a well-known risk factor for many chronic diseases, including chronic kidney disease (CKD) [1–3]. CKD tends to progress more rapidly in patients with OSA. Furthermore, OSA is more commonly diagnosed in patients with CKD. Bi-directional relationships between CKD and OSA have been frequently suggested [4, 5].

CKD may increase the risk of OSA by ventilatory instability, upper airway collapsibility, and possibly uremic myopathy, secondary to increased chemosensitivity, hypervolemia, and uremic toxins [6–9]. OSA may accelerate the progression of CKD by hypoxia, hypertension, increased sympathetic nervous system activity, and activation of the renin–angiotensin–aldosterone system [2, 10]. Because these noxious exposures to the kidney recur every night in patients with OSA, the damage to the kidney from nightly exposure to OSA may follow a circadian rhythm.

Glomerular filtration rate (GFR) changes from daytime to nighttime following the circadian rhythm. In healthy individuals, GFR drops at night, followed by daytime surge [11–13]. This normal diurnal variation is attenuated in patients with CKD as they often lose the nocturnal drop in GFR [14]. Because CKD can only be diagnosed when glomerular filtration rate has permanently declined, it may be advantageous to find early signs of kidney damage in high-risk patients before their GFR declines. We hypothesized that patients with severe OSA may lose the diurnal variation in GFR.

## Methods

From March 2021 to December 2021, we prospectively recruited participants (age ≥ 18 years) who were referred to the sleep clinic for habitual snoring or apnea during sleep. Anthropometric data and medical histories were obtained from the participants. Our exclusion criteria were a history of cardiovascular disease (stroke, coronal artery disease, and peripheral vascular disease) or known kidney disease.

The participants underwent in-laboratory polysomnography (PSG). They also underwent phlebotomy before and after the PSG to measure kidney biomarkers serum creatinine, blood urea nitrogen (BUN), and serum cystatin C. The participants were advised to avoid caffeine consumption, high-protein intake, or vigorous exercise within 24 h before the PSG. Blood samples for the kidney biomarkers were drawn in the evening between 3 p.m. and 5 p.m. preceding the PSG and in the morning between 7 a.m. and 8 a.m. following the PSG.

### In-laboratory polysomnography

All subjects underwent PSG with 24-h Holter monitoring. PSG was performed using a commercially available recording system (EmblaTM N7000; Embla, Reykjavik, Iceland). The standard electrodes and sensors, recommended by the American Academy of Sleep Medicine Manual (v2.4), were used to detect bio-signals from 11 p.m. to 7 a.m. under the supervision of a skilled technician. All sleep parameters were manually interpreted by the technician, according to the standard criteria of the 2017 American Academy of Sleep Medicine Manual (v2.4) for the Scoring of Sleep and Associated Events, and the data were reviewed by certified physicians. Apnea Hypopnea Index (AHI) is the total number of apneas and hypopneas per hour of sleep. Participants with AHI < 5 events/hr were categorized as simple snoring group and those with AHI ≥ 5 events/hr were diagnosed with OSA. The severity of OSA was further defined as follows: mild, 5 ≤ AHI < 15 events/hr; moderate, 15 ≤ AHI ≤ 30 events/hr; severe, AHI > 30 events/hr.

### Statistical analysis

Descriptive statistics are presented as percentages (categorical variables) or means ± standard deviations (normally distributed continuous variables). We used ANOVA to compare kidney biomarkers among the three groups with different severity of OSA. Pearson correlation coefficients were used to assess relationships between parameters of PSG and delta kidney biomarkers between the morning level and the evening level. A paired *t*-test was used to compare the morning kidney biomarkers to the evening biomarkers. All analyses were performed using SPSS version 18.0 software (IBM Corp, Armonk, NY), and a *P* value < 0.05 was considered statistically significant.

## Results

Fifty-six subjects were initially enrolled in the study. Four subjects were excluded based on the exclusion criteria. Additionally, two subjects were excluded from the study because they did not undergo morning phlebotomy, leaving a total of 50 subjects for final analysis. The 50 subjects were divided into three groups: simple snoring-to-mild OSA, moderate OSA, and severe OSA, based on their AHI as described in the Methods section. Of the 50 subjects, 26 were in the severe OSA group, which had the highest mean body mass index (BMI) of 29.2 ± 4.8 kg/m^2^ and the highest prevalence of hypertension at 38.5%. The severe OSA group also had the poorest quality of sleep parameters, including the least total sleep time (350.5 ± 112.5 min) and the least percentage of sleep stage N3 (8.5 ± 7.3%). Both the lowest O_2_ saturation (75.3 ± 1.0%) and mean O_2_ saturation (94.2 ± 2.8%) were the lowest in the severe OSA group (Table 1).

**Table 1:** Baseline characteristics of the participants

	Simple Snoring to Mild OSA (*n* = 12)	Moderate OSA (*n* = 12)	Severe OSA (*n* = 26)
Age, years	43.7 ± 15.8	48.5 ± 11.9	47.2 ± 12.3
Female, n (%)	2 (16.7)	4 (33.3)	3 (11.5)
BMI, kg/m^2^	24.9 ± 2.3	25.3 ± 3.4	29.2 ± 4.8
AHI, /hr	10.0 ± 4.6	20.9 ± 4.4	56.2 ± 23.4
ODI, /hr	5.7 ± 3.7	12.8 ± 4.0	45.7 ± 26.9
Snoring, %	29.2 ± 16.8	26.5 ± 20.4	47.7 ± 23.0
Total sleep time, min	407.9 ± 45.6	379.0 ± 50.5	350.5 ± 112.5
WASO, min	67.2 ± 36.5	82.9 ± 42.4	67.2 ± 43.8
Sleep efficiency, %	84.3 ± 8.1	77.7 ± 10.0	82.6 ± 8.6
Sleep stage N1, %	27.0 ± 14.0	23.8 ± 3.4	38.4 ± 15.5
Sleep stage N2, %	42.3 ± 7.3	41.8 ± 7.5	37.6 ± 11.2
Sleep stage N3, %	13.1 ± 10.1	12.9 ± 7.4	8.5 ± 7.3
Sleep stage REM, %	17.6 ± 5.9	21.7 ± 4.0	15.8 ± 6.5
Total arousal, /hr	24.5 ± 9.0	27.2 ± 7.6	52.1 ± 23.6
Mean O_2_ saturation, %	96.3 ± 1.2	96.1 ± 1.0	94.2 ± 2.8
Lowest O_2_ saturation, %	88.7 ± 4.1	85.3 ± 3.7	75.3 ± 1.0
Hypertension, n (%)	1 (8.3)	3 (25.0)	10 (38.5)
Diabetes mellitus, n (%)	0 (0.0)	2 (16.7)	2 (7.7)
Hyperlipidemia, n (%)	0 (0.0)	3 (25.0)	4 (15.4)
Current smoking, n (%)	1 (16.7)	4 (33.3)	7 (26.9)

The kidney biomarkers were compared between the three groups. No significant differences in BUN, creatinine, or cystatin C were noted between the three groups classified by OSA severity

*AHI* apnea–hypopnea index, *BMI* body mass index, *ODI* oxygen desaturation index, *OSA* obstructive sleep apnea, *WASO* wake after sleep onset

The level of BUN, creatinine, and cystatin C were compared between the three groups, but no significant differences were found (Table 2).

**Table 2:** The kidney biomarkers according to OSA severity

	Simple Snoring to Mild OSA (*n* = 12)	Moderate OSA (*n* = 12)	Severe OSA (*n* = 26)	*P* value
BUN, AM, mg/dL	14.5 ± 3.8	14.7 ± 2.9	14.7 ± 3.3	0.593
BUN, PM, mg/dL	15.9 ± 4.5	14.7 ± 2.0	14.5 ± 4.1	0.985
Cr, AM, mg/dL	0.88 ± 0.17	0.78 ± 0.17	0.84 ± 0.16	0.389
eGFR by Cr, AM^a^	104.3 ± 15.4	105.5 ± 12.4	104.4 ± 11.4	
Cr, PM, mg/dL	0.87 ± 0.15	0.79 ± 0.14	0.83 ± 0.16	0.447
eGFR by Cr, PM^a^	104.7 ± 13.5	105.8 ± 11.1	105.6 ± 11.5	
Cys, AM, mg/L	0.83 ± 0.12	0.85 ± 0.10	0.85 ± 0.11	0.823
eGFR by Cys, AM^a^	105.5 ± 15.7	98.6 ± 13.4	100.1 ± 13.1	
Cys, PM, mg/L	0.80 ± 0.12	0.81 ± 0.11	0.85 ± 0.10	0.377
eGFR by Cys, PM^a^	106.9 ± 16.7	102.0 ± 13.1	± 12.6	

*BUN* blood urea nitrogen, *AM* morning, *PM* evening, *Cr* creatinine, *Cys* cystatin C, *OSA* obstructive sleep apnea

^a﻿^ml/min/1.73m^2^

The delta kidney biomarkers for each participant were calculated by subtracting the evening level of biomarkers from the morning level. The delta cystatin C level was significantly correlated with AHI, ODI, and total arousal index, with coefficients of -0.314, -0.323, and -0.289, respectively. These results suggest that the subjects with less severe OSA had a greater increase in the morning cystatin C level compared with the evening cystatin C. Neither delta BUN nor delta creatinine was significantly correlated with any PSG parameters (Table 3).

**Table 3:** Pearson's correlation coefficient between change of biomarkers and PSG parameters

	ΔBUN	ΔCreatinine	ΔCystatin C
AHI	-0.150	0.050	-0.314*
ODI	-0.129	0.006	-0.323*
Mean O_2_ saturation	0.088	0.101	0.221
Lowest O_2_ saturation	0.123	-0.020	0.182
Total sleep time	0.156	0.191	0.087
Sleep efficiency	0.059	0.163	-0.118
Total arousal	-0.202	0.038	-0.289^*^

Δ, AM level—PM level, *BUN* blood urea nitrogen, *AHI* apnea–hypopnea index, *ODI* oxygen desaturation index, *O*_*2*_ oxygen

^*﻿^ *p* < 0.05

The subjects were also divided into two groups: those without severe OSA (AHI ≤ 30) and those with severe OSA (AHI > 30). The levels of BUN, creatinine, and cystatin C in each participant were compared between the evening and the morning. In the group without severe OSA, the morning cystatin C level (0.84 ± 0.11 mg/L) was significantly higher than the evening cystatin C level (0.81 ± 0.11 mg/L) (*P* = 0.005). In the severe OSA group, there was no significant difference in cystatin C levels between the evening (0.85 ± 0.10 mg/L) and the morning (0.85 ± 0.11 mg/L). No significant differences were observed in BUN or creatinine levels between the evening and morning in either group (Table 4).

**Table 4:** Diurnal variation of kidney biomarkers

	AM	PM	*P*-value
All participants (*n* = 50)			
BUN	15.0 ± 3.5	14.6 ± 3.6	0.360
Creatinine	0.84 ± 0.17	0.83 ± 0.15	0.641
Cystatin C	0.84 ± 0.11	0.83 ± 0.11	0.065
Without severe OSA (*n* = 24)			
BUN	15.3 ± 3.7	14.6 ± 3.0	0.386
Creatinine	0.83 ± 0.18	0.83 ± 0.15	0.890
Cystatin C	0.84 ± 0.11	0.81 ± 0.11	0.005
With severe OSA (*n* = 26)			
BUN	14.7 ± 3.3	14.5 ± 4.1	0.704
Creatinine	0.84 ± 0.16	0.83 ± 0.16	0.633
Cystatin C	0.85 ± 0.11	0.85 ± 0.10	0.841

*AHI* apnea–hypopnea index, *ODI* oxygen desaturation index, *BUN* blood urea nitrogen

**Fig. 1:**
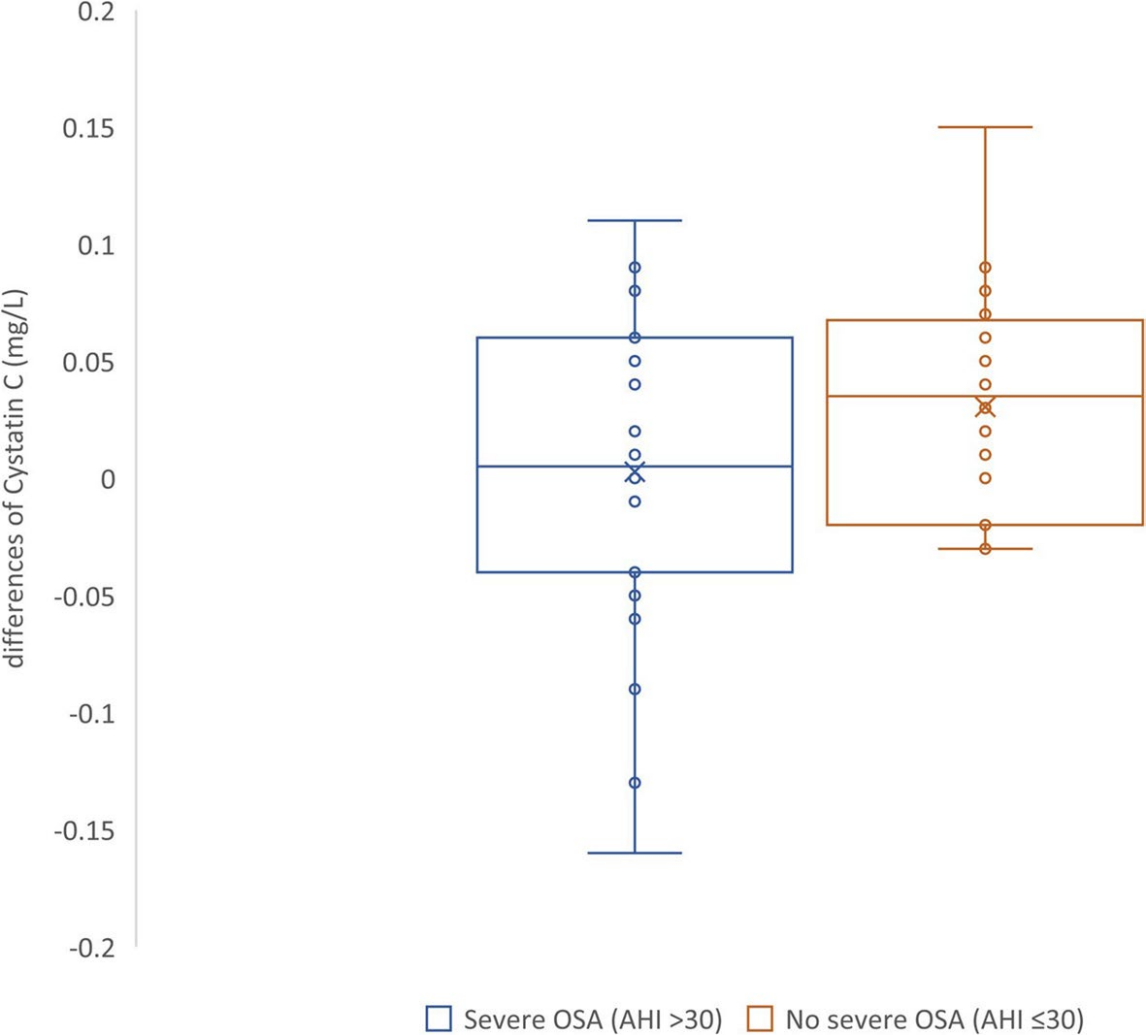
Differences in Cystatin C level Differences of cystatin C levels from the morning level to the evening level in each subject. The blue circles depict subjects with severe OSA (AHI >30) and the orange circles depict subjects without severe OSA (AHI ≤ 30). X marks denote the mean value for each group. OSA, obstructive sleep apnea; AHI, apnea-hypopnea index

In the group without severe OSA, the cystatin C level was significantly increased in the morning compared with that in the evening for many patients. However, this trend was often weakened or reversed in the participants with severe OSA (Fig. 1) (Supplemental Fig. 1).

## Discussion

CKD may increase the risk of OSA through several suggested mechanisms. Uremia in CKD can fatigue respiratory muscles or affect the sensory function of the upper airway, causing upper airway collapse [15]. Chemosensitivity that regulates ventilatory drive may be altered in CKD, resulting in ventilatory instability [8]. Fluid overload is a well-established risk for sleep apnea in patients with CKD, either by nasopharyngeal narrowing or by fluid accumulation in the lungs [9, 16].

OSA may accelerate progression of CKD by glomerular hyperfiltration and hypoxic injury to the kidney tubules and glomeruli. The association of hypoxia and kidney injury has been shown in both rat models and human subject studies [17, 18]. Glomerular hyperfiltration in OSA is mediated by nocturnal hypertension, activation of sympathetic nervous system, and activation of the renin–angiotensin–aldosterone system [2, 19].

In our study, for subjects without severe OSA, the serum cystatin C levels were significantly increased in the morning compared to the evening. In contrast, serum cystatin C levels did not significantly increase in the morning for those with severe OSA, suggesting the loss of nocturnal decline in GFR. No significant differences between morning and evening were observed in the level of BUN or serum creatinine. Individual dietary variation likely affected the level of BUN or serum creatinine, as their levels can vary based on the amount of daily protein intake. Therefore, BUN or serum creatinine is not a reliable kidney biomarker to assess the diurnal variation of GFR when the diet is not controlled among study participants [14].

Serum cystatin C has been widely used as a marker of GFR along with serum creatinine since Simonsen et al. demonstrated its close correlation to GFR in 1985 [20, 21]. It has been considered a comparable biomarker to serum creatinine in estimating GFR and predicting the risk of end-stage renal disease and major health outcomes [22]. Compared to creatinine, cystatin C is less affected by non-GFR determinants such as muscle mass, meat intake, age, and race [23]. Cystatin C can be particularly suitable in estimating GFR when the value is actively changing due to diurnal variation, as it has a shorter half-life (1.5 h), compared to creatinine (4 h) [24, 25].

Diurnal variation or circadian rhythm of GFR is a physiological finding. Koopman et al. showed average 30% change of GFR within 24 h in 11 healthy subjects, from its peak at 3 p.m. to its nadir at 3 a.m., by measuring their inulin clearances [11]. Their GFR declined at night and rebounded during the daytime, showing a circadian rhythm. The nocturnal decline of GFR dissipates for patients with CKD because the diseased kidney may continue to hyperfiltrate at night [14]. As is common in the pathophysiology of CKD, such as diabetic nephropathy, we often diagnose CKD later through elevated kidney biomarkers serum creatinine or cystatin C when the disease has already far progressed and irreversibly damaged the kidney. The subjects with severe OSA in our study presented no significant decline in their GFR at night, based on their cystatin C level. This phenomenon may be reminiscent of the observation in patients with CKD. For example, Hilderink et al. reported less prominent circadian rhythm of cystatin C in individuals with CKD [14]. Based on this, less prominent circadian rhythm in cystatin C levels coud be an early indicator of CKD progression in indivisuals with severe OSA. However, further studies are needed to confirm this speculation.

The nightly decline in GFR in subjects without severe OSA can be explained by physiological changes syncing with circadian rhythms, such as systemic blood pressure, renal blood flow, regulation of afferent and efferent arteriolar resistance by the renin–angiotensin–aldosterone system, prostaglandin E2, and antidiuretic hormone [12]. Above all, nightly dipping in blood pressure in healthy individuals is a well-known physiological change with a circadian rhythm and is significantly associated with the nightly decline in GFR [26, 27]. Therefore, nocturnal hypertension, which is prevalent in patients with severe OSA, may have contributed to the elevated nightime GFR in the severe OSA group [28, 29]. Sleeping itself was an unlikely contributor to the circadian rhythm of GFR in a previous study. Larsson et al. showed that acute shift to daytime sleeping from nighttime sleeping did not alter the circadian rhythm of GFR [30].

Our study has the following strengths. We attempted to assess early signs of chronic kidney disease by assessing diurnal variation of GFR in patients with severe OSA, whereas the exact mechanism of CKD progression in patients with severe OSA is still poorly understood and early screening tools are absent until their GFR declines permanently. Although we have not proven that loss of the diurnal variation or the nightly drop in GFR is associated with CKD progression, the association is plausible because such loss was previously observed in patients with CKD [14].

Our study has weaknesses. We failed to measure other important variables which might alter the diurnal variation of GFR, such as systemic blood pressure and associated hormones renin, angiotensin, epinephrine, norepinephrine, or antidiuretic hormone. We included patients on the medications that may affect GFR, including angiotensin-converting-enzyme inhibitors, angiotensin receptor blockers, or diuretics. Previous studies have shown that these medications may cause nocturnal dipping in systemic blood pressure for patients with nocturnal hypertension [31, 32]. Therefore, for subjects who would normally have nocturnal hyperfiltration, these medications may have caused a nocturnal decline in GFR instead. We used serum cystatin C level to estimate GFR, which is imperfect. Although serum cystatin C level is less influenced by diet or muscle mass than serum creatinine level, it is inferior to methods that directly measure GFR with exogenous molecules, such as inulin, iothalamate, or diethylenetriaminepentaacetic acid.

Patients with OSA are at a high risk of CKD, yet we do not have methods to screen early signs of kidney damage until their GFR declines permanently. The absence of nighttime drop in GFR, secondary to nocturnal hyperfiltration, in patients with severe OSA may be an early sign of CKD progression. Future studies can be designed to elucidate the correlation between diurnal variation of GFR and progression of CKD.

### Supplementary Information

**Additional file 1:  Supplemental Figure 1.** Diurnal variation of cystatin C level in each subject. The left figure depicts change in cystatin C level for subjects without severe OSA (AHI ≤ 30). The right figure depicts the change for those with severe OSA (AHI > 30). Cystatin C level is mg/L. PM, evening level; AM, morning level.

## Data Availability

The datasets generated or analysed during the current study are available from the corresponding author on reasonable request.
