# Supplementary material for: Association of obstructive sleep apnea and diurnal variation of cystatin C
Source: BMC Nephrol. 2024 Jan 29;25:40. doi: 10.1186/s12882-024-03472-7 (PMC10823701; doi:10.1186/s12882-024-03472-7)
Supplement: Supplementary file 1 — Additional file 1: Supplemental Figure 1. Diurnal variation of cystatin C level in each subject. The left figure depicts change in cystatin C level for subjects without severe OSA (AHI ≤ 30). The right figure depicts the change for those with severe OSA (AHI > 30). Cystatin C level is mg/L. PM, evening level; AM, morning level. [file 12882_2024_3472_MOESM1_ESM.docx]

**
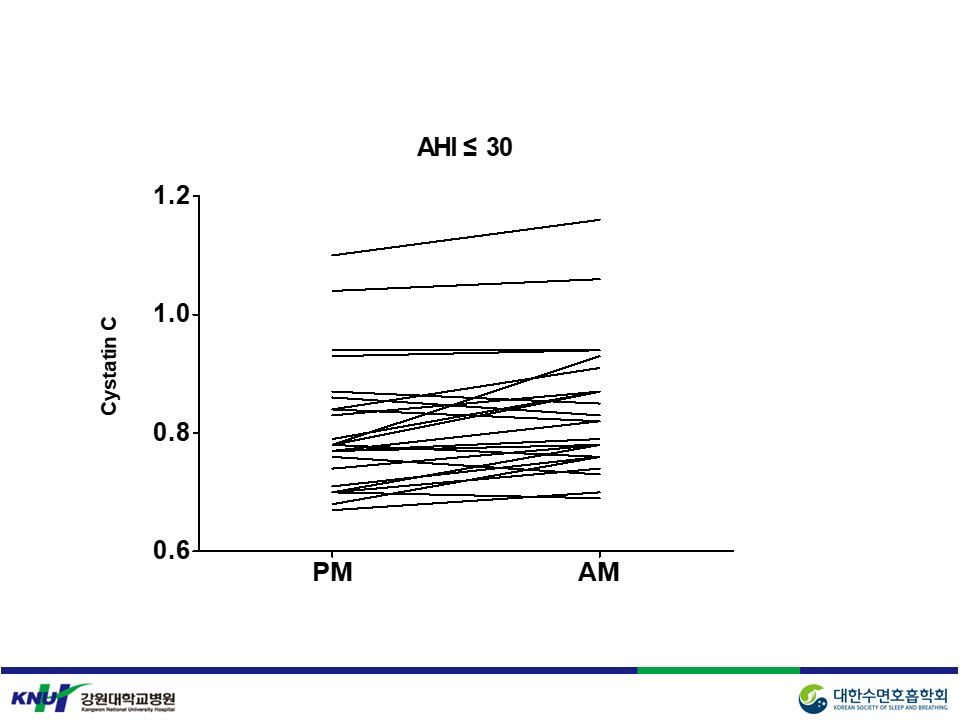

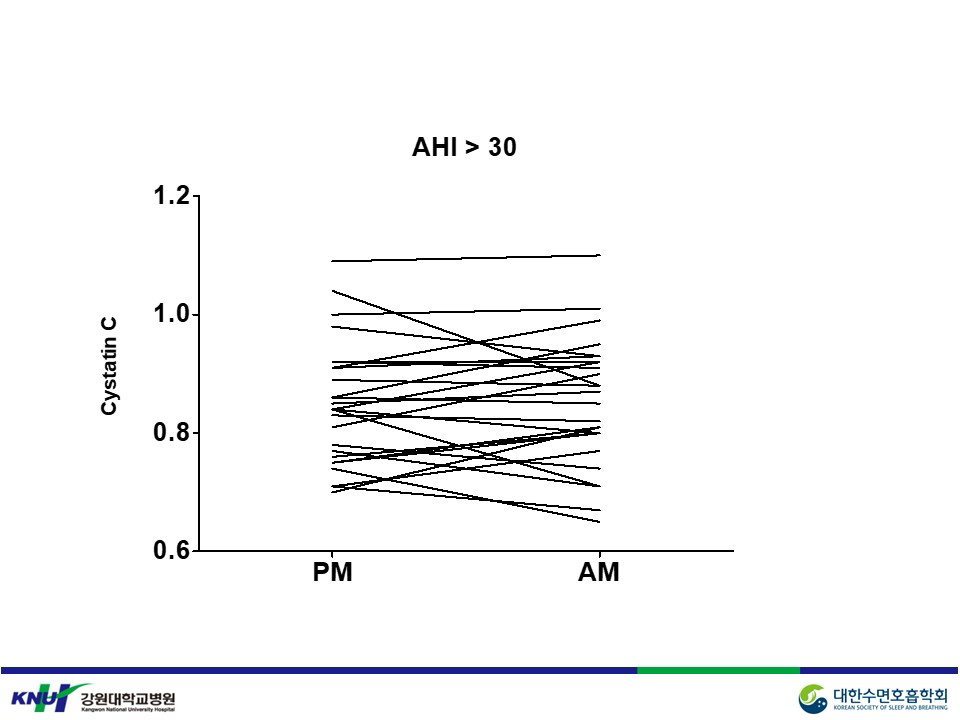
**

**Supplemental Figure 1.** **Diurnal variation of cystatin C level in each subject.** The left figure depicts change in cystatin C level for subjects without severe OSA (AHI ≤ 30). The right figure depicts the change for those with severe OSA (AHI > 30). Cystatin C level is mg/L. PM, evening level; AM, morning level.
